# Alliin, a Garlic (*Allium sativum*) Compound, Prevents LPS-Induced Inflammation in 3T3-L1 Adipocytes

**DOI:** 10.1155/2013/381815

**Published:** 2013-12-26

**Authors:** Saray Quintero-Fabián, Daniel Ortuño-Sahagún, Manuel Vázquez-Carrera, Rocío Ivette López-Roa

**Affiliations:** ^1^Laboratorio de Desarrollo y Regeneración Neural, Instituto de Neurobiología, Departamento de Biología Celular y Molecular, CUCBA, Universidad de Guadalajara, Camino Ing. R. Padilla Sánchez 2100, Las Agujas, 44600 Zapopan, JAL, Mexico; ^2^Departamento de Farmacobiología, CUCEI, Universidad de Guadalajara, Boulevard Marcelino García Barragán, No. 1421, Esquina Calzada Olímpica, 44430 Guadalajara, JAL, Mexico; ^3^Unidad de Farmacología, Departamento de Farmacología y Química Terapéutica, Facultad de Farmacia, Institut de Biomedicina de la UB (IBUB) and CIBER de Diabetes y Enfermedades Metabólicas Asociadas (CIBERDEM)-Instituto de Salud Carlos III, 08028 Barcelona, Spain

## Abstract

Garlic (*Allium sativum* L.) has been used to alleviate a variety of health problems due to its high content of organosulfur compounds and antioxidant activity. The main active component is alliin (S-allyl cysteine sulfoxide), a potent antioxidant with cardioprotective and neuroprotective actions. In addition, it helps to decrease serum levels of glucose, insulin, triglycerides, and uric acid, as well as insulin resistance, and reduces cytokine levels. However its potential anti-inflammatory effect is unknown. We examined the effects of alliin in lipopolysaccharide- (LPS-) stimulated 3T3-L1 adipocytes by RT-PCR, Western blot, and microarrays analysis of 22,000 genes. Incubation of cells for 24 h with 100 **μ**mol/L alliin prevented the increase in the expression of proinflammatory genes, IL-6, MCP-1, and Egr-1 in 3T3-L1 adipocytes exposed to 100 ng/mL LPS for 1 h. Interestingly, the phosphorylation of ERK1/2, which is involved in LPS-induced inflammation in adipocytes, was decreased following alliin treatment. Furthermore, the gene expression profile by microarrays evidentiate an upregulation of genes involved in immune response and downregulation of genes related with cancer. The present results have shown that alliin is able to suppress the LPS inflammatory signals by generating an anti-inflammatory gene expression profile and by modifying adipocyte metabolic profile.

## 1. Introduction

Obesity has been traditionally linked to metabolic dysfunction, led by adipocyte proliferation and hypertrophy [1]. However, some other systemic alterations are being studied as related to obesity, for example, inflammation and immune dysfunction [2]. Adipose tissue is now widely considered as an endocrine tissue capable of producing chronic inflammatory responses [3–5]. Obesity has been shown to cause an increase in plasma concentrations of a number of proinflammatory markers (e.g., IL-6, TNF-*α*) that are expressed and released by adipocytes [6]. Diet-induced obesity increases local and systemic inflammatory adipocytokines in humans and in rodents; these factors contribute to adverse health outcomes [7]. The homeostatic balance between pro- and anti-inflammatory cytokines and adipokines defines the profile and magnitude of inflammation and its effects on insulin sensitivity and glucose homeostasis [8]. Therefore, therapies able to modulate the inflammatory state of adipose tissue are being considered for the treatment of obesity [9]. However, factors that might mitigate or act against this inflammatory response have remained elusive.

Garlic (*Allium sativum*) is one of the oldest medicinal plants used by different cultures [10]. Allium vegetables comprise one natural source of organic sulfur-containing compounds and have been widely investigated regarding their therapeutic applications [11, 12], mainly due to its cardioprotective effect and to its anticancerogenic properties [10, 13]. However, its anti-inflammatory effects have received less attention. For example, garlic compounds can exert an anti-inflammatory effect by inhibiting the oxidative stress-induced activation of nuclear factor-kappa B (NF-*κ*B) [14], which is implicated in the expression of proinflammatory enzymes such as inducible nitric oxide synthase (NOS) and cyclooxygenase-II (COX-II). In particular, allicin and ajoene, two garlic compounds, significantly suppressed nitric oxide production by lipopolysaccharide (LPS) stimulation, accompanied by suppression in inducible nitric oxide (iNOS) expression and iNOS activity [15]. Although it appears promising that garlic and its derivatives possess antidiabetic potential, an understanding of the antidiabetic effects of garlic is still in its early stages. Furthermore, the active compounds in garlic, and doses thereof, which can effectively provide antidiabetic effects (i.e., glycemic control and amelioration of diabetic complications) remain to be established.

Alliin (S-allyl cysteine sulfoxide), first identified by Stoll and Seebeck in 1947, is considered the main specific principle of garlic since that time [16]. Alliin can be found in intact garlic and its reduced form; S-allyl-cysteine is the major component of aged garlic extract (AGE), together with other derived organosulfur compounds [12, 13]. It is absorbed in the intestine via the amino acid transported for cysteine [17], exhibits an hypoglycemic effect, and also increases blood insulin concentrations [18], and its antioxidant activity has been widely studied [19]. Nonetheless, to our knowledge, its anti-inflammatory potential has not yet been explored.

Mouse preadipocyte 3T3-L1 cells are the most commonly studied and available adipogenic cell line. This cell line has been quite useful in identifying key molecular markers, transcription factors, and various interactions that are required for preadipocyte differentiation [20]. Additionally, 3T3-L1 adipocytes are able to respond to LPS by means of a fully intact innate immunity pathway, through the production and secretion of immunomodulatory (mainly proinflammatory) molecules such as IL-6, TNF-*α*, and TLR-2 [21], and through a TLR-ligand-induced activation, which triggers a proinflammatory and -diabetic transformation of adipocytes [22]. Interestingly, this immune response activation is resembled by human adipose tissue [23]; consequently, once differentiated, 3T3-L1 appears to be a suitable model to test the anti-inflammatory response that affects the metabolic regulation of adipocytes elicited by chemical compounds and nutraceutics under controlled conditions [24–27]. Thus, to determine the anti-inflammatory effect of alliin, in the present work we used the LPS-stimulated 3T3-L1 cell line as an inflammatory model* in vitro*. Our findings indicate that alliin prevents LPS-induced inflammation by inhibiting ERK1/2 in 3T3-L1 adipocytes.

## 2. Materials and Methods

### 2.1. Cell Culture

3T3-L1 cells were obtained from American Type Culture Collection (ATCC CL-173) and cultured at 37°C in 5% CO_2_/95% humidified air. The cells were maintained in Dulbecco's Eagle modified medium (DMEM) with 25 mM HEPES, 1% penicillin-streptomycin (100 U/mL; 100 *μ*g/mL), and 10% calf serum. The cells were differentiated 2 days after confluence (day 0) in DMEM containing 10% Fetal bovine serum (FBS), 0.25 *μ*M dexamethasone, 0.5 mM 3-isobutyl-1-methylxanthine, and 5 *μ*g/mL insulin for 48 h; the cells were then incubated in 10% FBS/DMEM with insulin for 72 h. Finally 10% FBS/DMEM was changed every 2 days.

### 2.2. T3-L1 Cell Treatment

On day 8 after differentiation, the cells were pretreated during a 24 h period with a medium containing 0.1 mM alliin (Sigma Chemical Co.). Cells were then incubated with the same medium along with LPS (100 ng/mL) for 1 h before being harvested. The cultured medium was collected in tubes and stored at −80°C. Preliminary experiments were performed to determine the concentrations of alliin during time-course experiments (data not shown).

### 2.3. mRNA Expression Quantification by qPCR

Total RNA was isolated from cells by using the Ultraspec reagent (Biotecx, Houston, TX, USA). First-strand cDNA was reverse-transcribed from 0.5 *μ*g of total RNA by employing the M-MLV Reverse Transcriptase protocol (Invitrogen). The cDNA was amplified with different pairs of specific primers that were designed utilizing *PrimerExpress* software (Table 1).

The real-time quantitative PCR reaction contained 0.2 *μ*g/*μ*L of reverse-transcribed total RNA, 18 *μ*M forward and reverse primers, and 10 *μ*L of iQ SYBR Green SuperMix (Bio-Rad). PCR was performed in 48-well plates with the MiniOpticon real-time PCR system (Bio-Rad). Quantification was performed by the comparative cycle of threshold method, with the invariable adenine phosphoribosyltransferase (*Aprt*) gene used for normalization.

### 2.4. Protein Analysis by Western Blotting

To obtain total protein from 3T3-L1 adipocytes, cells from different treatments were homogenized in 50 *μ*L RIPA buffer at 4°C supplemented with 10 mM sodium orthovanadate, 100 mM PMSF, and 5.4 mg/mL aprotinin. Insoluble material was removed by centrifugation for 30 min at 13,500 g at 4°C. The protein concentration of the supernatants was determined by the Bradford method. Proteins were denatured by boiling (5 min); then 30 *μ*g were resolved by SDS-PAGE on 10% separation gels and transferred to immobilon PVDF membranes (Millipore, Bedford, MA, USA). Nonspecific protein binding to the PVDF membrane was reduced by preincubation for 1 h at 22°C in TBS tween-20 (0.1%) and 5% bovine serum albumin (BSA) blocking buffer. The PVDF membranes were incubated overnight at 4°C with monoclonal antibodies against total and phosphorylated ERK1/2 (Cell Signaling and Santa Cruz Biotechnology, Santa Cruz, CA, USA), which were diluted 1 : 1,000 in blocking buffer. After incubation, the membranes were washed for 5 min three times in TBS tween-20 (0.1%). The blots were subsequently incubated with peroxidase-conjugated secondary antibody for 1 h at 22°C in TBS tween-20 (0.1%) and 5% (w/v) fat-free milk. For evaluation of protein loading, the membranes were incubated and reblotted with anti-*β*-actin antibody (Santa Cruz Biotechnology) as appropriate. Specific bands were detected using the EZ-ECL chemiluminescence kit (Amersham), and visualization/capture was performed by exposure of the membranes to RX films. Band intensities were quantified by Quantity One software (Bio-Rad).

### 2.5. Analysis of Cytokine Levels by Enzyme-Linked Immunosorbent Assays (ELISA)

Cytokines and chemokines were measured in cell culture supernatants using ELISA techniques (Bio-Plex Pro Assays; Bio-Rad Laboratories, Inc.). The lower detection limit was 38 pg/mL for IL-6, 21 pg/mL for TNF-*α*, and 51 pg/mL for MCP-1. For data normalization and to exclude artificial effects of different cell densities, total protein concentration was measured for each well, and supernatant cytokine concentrations were divided by protein concentration. Each sample was measured in triplicate by ELISA. Values were expressed as mean ± standard error of the mean (SEM) (*n* = 6 wells were used for each experimental group).

### 2.6. Printing of Arrays, Probe Preparation, and Hybridization of Arrays

A *Mus musculus* 22,000 65-mer Oligo Library from Sigma-Genosys sets was used. For the hybridization experiments, the RNA utilized was from cells collected from cultures. For cDNA synthesis, 10 *μ*g of total RNA was used as the template, incorporating dUTP-Cy3 or dUTP-Cy5, and equal quantities of labeled cDNA were hybridized to the 22,000 oligo mouse arrays, as described previously [28, 29].

### 2.7. Data Acquisition and Analysis of Array Images

Acquisition and quantification of the array images were performed in a ScanArray 4000 apparatus using the accompanying software ScanArray 4000 (Packard BioChips; Perkin-Elmer, MN, USA). All images were captured as described previously [28, 29]. In all cases, the fluorescence signal was from seven to ten times more intensive than the background signal, and the background evaluation was always evaluated immediately beside the labeled spot.

### 2.8. Data Analysis

Microarray data analysis was performed using GenArise free software. The goal of GenArise is to identify genes that are good candidates for differential expression by calculating an intensity-dependent *z*-score, successfully used and recently reported [28, 29]. Applying these criteria, the elements with a *z*-score of >2 standard deviations (SD) are genes likely to be differentially expressed.

### 2.9. Statistical Analysis

The results are expressed as the mean ± SEM. For statistical analysis, GraphPad Prism software was employed and the one-way ANOVA test was applied to compare the treatment effects. Significant differences were established by the Turkey-Kramer test. Values of *P* < 0.05 were considered statistically significant.

## 3. Results

### 3.1. Alliin Pretreatment Significantly Reduces the mRNA Expression and Protein Levels of Proinflammatory Molecules IL-6 and MCP-1 after LPS Exposure in 3T3-L1 Adipocytes

Previously, we determined the alliin concentration that exerts an effect on the expression of the tested genes; the concentrations probed were 0.1, 0.3, 0.6, and 1.0 mM (data not shown). From this we selected 0.1 mM as the minimum concentration able to elicit a clear effect.

**Figure 1:**
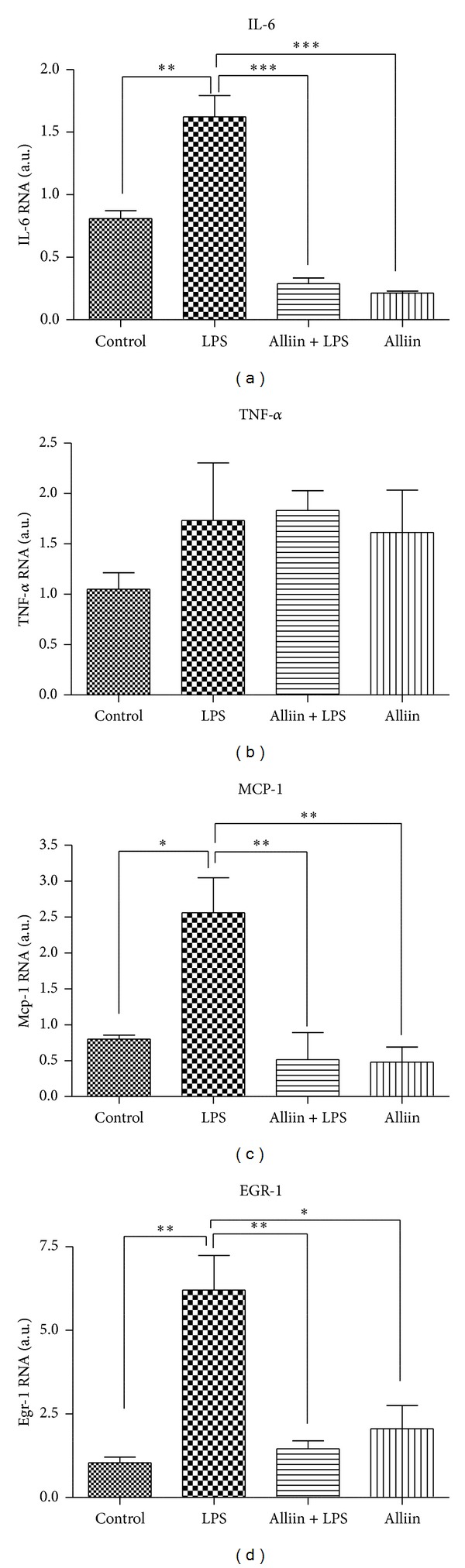
Messenger RNA (mRNA) expression levels of proinflammatory genes. Differentiated adipocytes were incubated with 0.1 mM/mL alliin for 24 h and stimulated with 100 ng/mL of lipopolysaccharides (LPS) for 1 h. Values are expressed as arbitrary units (AU) after normalization of expression levels against a control gene. Results are mean ± standard deviations (SD) of three independent experiments. *P ≤ 0.05; **P ≤ 0.01; ***P ≤ 0.001.

Cytokine IL-6 is correlated with insulin resistance in subjects with obesity and is inducible through TLR-4 receptor activation [30]. After the alliin pretreatment, mRNA levels for IL-6 were significantly reduced (Figure 1(a)). In contrast, the level of TNF-*α* mRNA was apparently not significantly affected, although a slight tendency toward its decrease in alliin pretreated cells was also noted (Figure 1(b)).

Additionally, we checked for MCP-1 expression because it is produced by a variety of cells, including adipocytes, in response to inflammatory stimuli [31]. As expected, we found a significant increase in MCP-1 expression in LPS-treated adipocytes. Interestingly, we again observed a significant reduction in MCP-1 mRNA levels when LPS-stimulated adipocytes were pretreated with alliin (Figure 1(c)).

Furthermore, we verified the expression of Egr-1, which is described as induced by cytokines and hormones through activation of the MAPK pathway and which are related with insulin resistance [32]. Once again, the mRNA expression level was significantly reduced by alliin pretreatment even after the LPS proinflammatory stimulus (Figure 1(d)).

**Figure 2:**
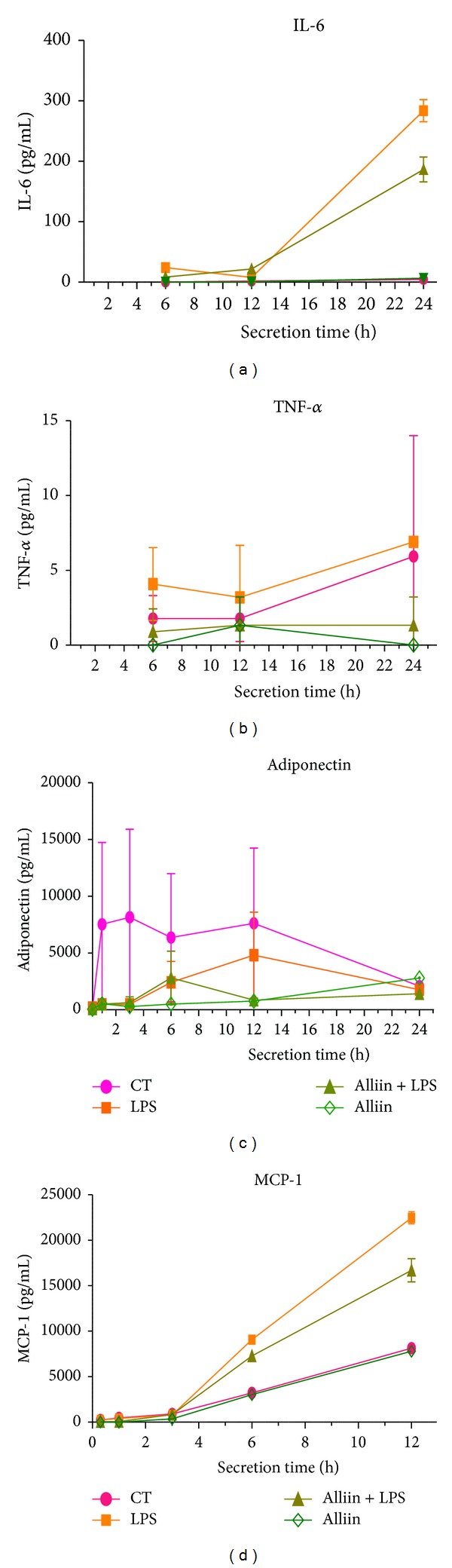
Protein expression levels of proinflammatory and anti-inflammatory proteins secreted by 3T3-L1 adipocytes. Cells were incubated with 0.1 mM alliin for 24 h and exposed to 100 ng/mL of lipopolysaccharides (LPS) for 1 h. Cytokine and protein concentration in cell culture supernatants for 30 min, 1, 3, 6, 12, and 24 h after LPS exposure were determined by Luminex technology. Values are expressed in pg/mL of supernatant. Results are mean ± standard deviations (SD) of three independent experiments. *P ≤ 0.05; **P ≤ 0.01; ***P ≤ 0.001.

To corroborate these results, we evaluated the secreted protein levels of these cytokines and determined their release into the culture media by ELISA. Protein levels detected after the LPS stimulus, which are significantly reduced by alliin pretreatment, are shown in the case of IL-6 (Figure 2(a)) and Mcp-1 (Figure 2(d)). Moreover, we observed a reduction in TNF-*α* levels (Figure 2(b)), although this was small and did not reach statistical significance. Additionally, we tested for adiponectin levels (Figure 2(c)) because this represents an important union between obesity and insulin resistance and is considered as an anti-inflammatory protein [33]. The control group of adipocytes secretes a large amount of adiponectin (Figure 2(c)), which is clearly reduced by LPS stimuli. In the group pretreated with alliin, a slight increase can be observed in the production of this protein; however, it cannot overcome the severe reduction elicited by LPS.

### 3.2. Alliin Exerts Its Anti-Inflammatory Effect at Least through Diminishing the Phosphorylation of ERK1/2

**Figure 3:**
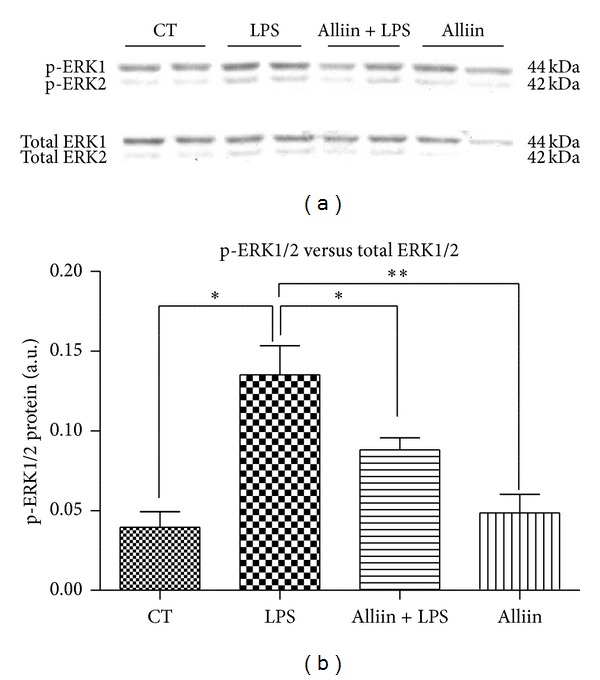
Levels of phosphoextracellular signal-regulated kinase (ERK1/2 p44/p42) in mouse 3T3-L1 adipocytes. Cells were pretreated for 24 h with alliin 0.1 mM and subsequently exposed to 100 ng/mL of lipopolysaccharides (LPS) for 1 h afterward. (a) Representative Western blot with phospho-ERK1/2 and ERK1/2 antibodies; (b) protein levels of phospho-ERK1/2 and ERK1/2 in total cell extracts. CT control; AU arbitrary units. Data are expressed as mean ± standard deviations (SD) of three independent experiments *P ≤ 0.05; **P ≤ 0.01.

Since LPS induces inflammation in adipocytes through ERK1/2 [30] and IL-6 and Egr-1 intracellular signaling mechanisms converge in this pathway, we next examined whether alliin pretreatment affects ERK1/2 phosphorylation. LPS stimulus is able to increase the protein levels of phosphorylated ERK1/2, and alliin pretreatment overwhelms this effect by significantly reducing this level, to nearly reach control levels (Figures 3(a) and 3(b)).

### 3.3. Gene Expression Profile of Alliin Pretreated 3T3-L1 Cells after LPS Stimulus Is Consistent with a Shift in Cell Response to Inflammatory Stimulus and Reveals Alliin Action on Adipocyte Physiology

Given that many other molecules can be involved both in the LPS inflammatory effect and also in the anti-inflammatory effect of alliin in this model, we decided to perform a microarray analysis to identify other genes involved in both effects. After analysis of the microarrays by GeneArise software, we found that of a total of the 22,000 genes analyzed in the microarrays, a total of 2,426 genes (11%) modify their expression, with a *z*-score between 2 and 6.2 in at least one of the three comparisons performed, which were the following: control versus LPS; control versus alliin + LPS, and LPS versus alliin + LPS. The remainder did not exhibit significant variations in expression, obtaining a *z*-score of <±2.0.

Comparison versus control group (nonstimulated adipocytes), LPS treatment, with or without alliin pretreatment, clearly modifies the expression of 315 genes (upregulating 255 and downregulating 60 genes) in common between the two comparisons (control versus LPS and control versus alliin + LPS). When analyzed by DAVID software [34] at high astringency, 237 of 315 (75%) correspond to identified genes, which can be grouped into 64 gene clusters according to functional cluster analysis. This profile is totally consistent with dozens of previous reports regarding the effect of LPS on the gene expression of numerous genes. The clusters that reach an enrichment score of >1.5 according to DAVID analysis [34], as well as representative genes whose expression is significantly modified by LPS in 3T3-L1 differentiated adipocytes, independently of the alliin pretreatment can be consulted in Table 1-SM (See Table 1-SM in Supplementary Material available online at http://dx.doi.org/10.1155/2013/381815). Therefore, as a first conclusion, the 315 genes detected here as modified by LPS confirms the effect of LPS and also enlarges the panorama concerning the effect of LPS on differentiated adipocytes *in vitro*, including several new genes whose mRNA expression are most probably affected by LPS proinflammatory stimuli.

On the other hand, we found 2,108 genes that were modified by alliin action after proinflammatory LPS stimuli. Of these, a subgroup of 125 genes was modified in at least two of the comparisons performed (54 were upregulated and 71 were downregulated with a *z*-score > 2.0); thus we can consider these genes as those whose expression is modified by alliin pretreatment. When analyzed by DAVID software [34] at high astringency, 100 of these genes were identified (80%) and grouped into 23 functional clusters. These functional clusters can be regrouped into nine functional categories by obvious similarities in cluster function. Data can be consulted in Table 2-SM.

Based on previous works [28, 29], we further conducted a more detailed analysis of the genes that reach a median *z*-score of >2.5 (up- or downregulated) between two group comparisons. We propose that genes fitting these criteria are those with greatest relevance and whose expression level is modified by alliin pretreatment in response to proinflammatory stimuli. All of the genes that were modified by alliin pretreatment in a sole comparison or with a median *z*-score of between 2.0 and 2.5 were excluded from this analysis. With this high-stringency criteria, we obtain 28 genes upregulated by alliin and 35 that were downregulated (full results can be consulted in Tables 3-SM and 4-SM). When individual analyses were performed, among the upregulated genes, we found that the most relevant group included 10 genes (36%) and is related with immunoglobulin and the T-cell receptor. The second relevant group includes eight genes than encode for different enzymes, and finally there was a third group of eight diverse genes and two additional unidentified genes (Table 2). Among the genes downregulated by alliin, we identified a main group that includes 15 genes that are cancer-related (Table 3), in addition to a group of five genes that encodes for enzymes, and a third group of seven genes related with diverse functions. It is noteworthy that a fourth group includes 10 unknown genes, which represent 29% of genes downregulated by alliin.

Thus, we present here the gene expression profile elicited after LPS stimuli when adipocytes receive an alliin pretreatment. These results provide large and clear evidence supporting alliin action, which clearly reverses the proinflammatory effect of LPS, as well as performing other parallel effects, because there is a clear shift in the gene expression profile.

## 4. Discussion

Because obesity is the result of a complex combination of multiple genetic and environmental factors, the consumption of certain nutraceuticals, such as garlic components, instead of or even in addition to highly energetic, fatty, and palatable foods, is able to modulate the metabolic functioning of adipose tissue in a way that results in the attenuation of the inflammatory state, therefore improving the quality of life [35].

### 4.1. Inflammatory Induction by LPS in 3T3-L1 Is Counteracted by Alliin Treatment

Differentiated adipocytes are equipped with the capability to respond directly to innate immune challenge by LPS from Gram-negative bacteria [21, 23, 36]. Some studies have shown the relevance of 3T3-L1 adipocytes, fully differentiated *in vitro* and stimulated by LPS, as a useful model to test for molecules that exhibits an anti-inflammatory effect and that are able to modulate the inflammatory state of adipose tissue [37]. LPS causes induction of IL-6 production, upregulation of TLR-2, and a downregulation of adiponectin receptors 1 and 2 [36]. Multiple signaling pathways mediate LPS induction of IL-6 [38, 39]. These pathways include NF-*κ*B, c-JNK, ERK, inhibitory G protein, and PKC mediated processes, and Toll-like receptors activate similar but distinct signaling pathways due to their ability to recruit different adapter proteins [22].

**Figure 4:**
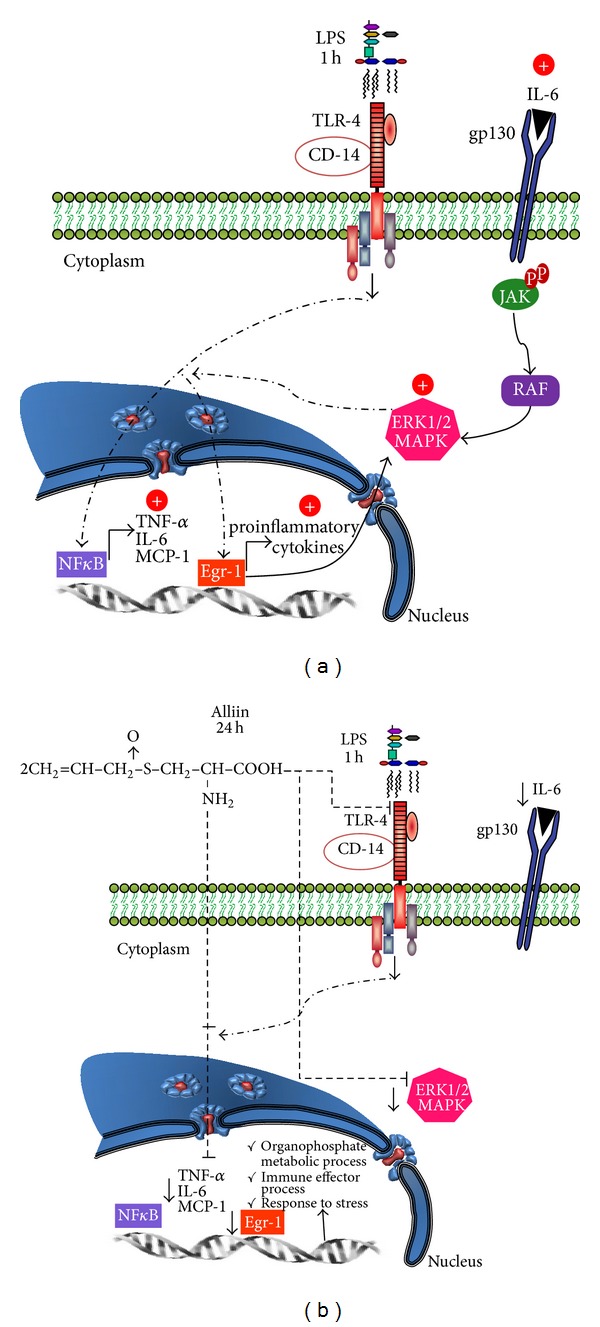
A proposal of how alliin could counteract the inflammatory state promoted by lipopolysaccharides (LPS) in 3T3-L1 adipocytes. (a) Activation of proinflammatory signaling pathway by lipopolysaccharides (LPS). (b) Alliin could reduce the Toll-like receptor-4 (TLR-4) pathway, possibly by diminishing the expression of related genes and proteins such as interleukin-6 (IL-6), monocyte chemostatic protein-1 (MCP-1), and early growth receptor-1 (Egr-1) and therefore regulates extracellular signal-regulated kinase (ERK1/2) activity.

LPS induces lipolysis in adipocytes via TLR-4 and ERK1/2 signaling [40]. Regarding TLR-4, this receptor recognizes LPS as a ligand [41] and it was demonstrated that stimulation of adipocytes by LPS attenuates insulin signaling by stimulating NF-*κ*B signaling, decreasing AKT phosphorylation, and increasing the expression of inflammatory cytokine genes such as TNF-*α* and IL-6 in 3T3-L1 adipocytes [42], which implicate this receptor in the onset of insulin resistance in obesity and type 2 diabetes. On the other hand, ERK1/2 activation is crucial for the induction of inflammatory changes in adipocytes [43] and leads to enhanced NF-*κ*B activity [44]. Activation of PPAR-*β*/*δ* inhibits enhanced cytokine production in adipocytes by preventing NF-*κ*B activation via ERK1/2, an effect that may aid in preventing insulin resistance [30]. Additionally, stimulation of 3T3-L1 adipocytes by LPS induces adipocytic insulin resistance via the involvement of JNK [45]. Therefore, ERK1/2 signaling and JNK signaling are relevant in adipose tissue physiology for the regulation of insulin sensitivity and lipolysis [40, 45]. On the basis of all of this background, and taking into account the results presented here, it is reasonable to propose one of the possible molecular mechanism by which alliin protects against the LPS effect (Figure 4).

Among factors that might mitigate the inflammatory response in adipocytes, it has been demonstrated that pretreatment of adipocytes with adiponectin exerts an anti-inflammatory activity by suppressing IL-6, TNF-*α* and MCP-1 production from inflamed adipocytes [46, 47]. This anti-inflammatory action may be mediated through inhibition of NF-*κ*B activity, as well as through increased PPAR-*γ* expression. Recently, Gómez-Arbeláez et al. showed that AGE is able to improve adiponectin levels in patients with metabolic syndrome after 12 weeks [48]. Here we show that a 24 h pretreatment of alliin is able to partially recover adiponectin levels in LPS-stimulated adipocytes. This further supports the anti-inflammatory effect of alliin.

In summary, we found that pretreatment with alliin counteracted LPS-induced inflammation. Our findings show that alliin downregulates IL-6 and MCP-1 expressions, as well as ERK1/2 phosphorylation. On this basis, our data indicate that speculation can ensue concerning that one of the alliin effects is caused probably through the NF-*κ*B system, as a signaling pathway used to suppress cytokine production in adipocytes (Figure 4). However, this proposed mechanism does not completely eliminate other possibilities such as, for example, the reduction of inflammation through the, already described, antioxidant effect of alliin, which can function as a complementary pathway.

### 4.2. Gene Expression Profile by Microarrays Induced by LPS Affects Several Groups of Genes and This Effect Is Counteracted by Alliin

Yamashita et al. [49] have showed that when stimulated with LPS, 3T3-L1 adipocytes cocultured with murine macrophages, these upregulated the expression of genes associated with inflammation, insulin resistance, and angiogenesis. The authors analyzed the gene expression profile of only 5,693 genes. Here we analyzed the gene expression profile of 22,000 genes after LPS stimuli with or without preincubation with alliin; thus our results extend the gene expression profile of 3T3-L1 adipocytes elicited by LPS stimulation, which is consistent with the regulation of the expression genes related with response to peptide hormone stimulus, cytoplasmic membrane-bounded vesicle, DNA/RNA helicase, DEAD/DEAH box type, ATP-binding, positive regulation of phagocytosis, regulation of vesicle-mediated transport, and ATP-dependent helicase activity, as the main clusters represented in the analysis (Table 1-SM).

We show here the gene expression profile induced by alliin pretreatment in response to LPS inflammatory stimuli (Tables 2 and 3). When we analyzed in detail the genes upregulated by alliin pretreatment with a higher median *z*-score (>2.5) in two comparisons, we found 10 genes related with immunoglobulin production and with T-cell receptors (Table 2), suggesting that alliin could exert an effect on the 3T3-L1 adipocytes inducing lymphopoietic activities. This is highly relevant in the context of the chronic proinflammatory state in obesity that is induced by adipocytes and that affects T-cell activation [50]. The expression of immunoglobulin receptors by adipocytes has already been mentioned in part [51, 52]; however, it has received nearly no attention. Here we show that alliin induces a differential expression of six immunoglobulin-related genes and four T-cell receptor-related genes in 3T3-L1 adipocytes. These immunoglobulins could act as anti-inflammatory signals or could at least influence adipocyte metabolism and, together with T-cell receptor genes, could affect the signaling between adipocytes and T cells.

It is also relevant that alliin appears to upregulate Cnpy4 (PRAT4B) expression, because this molecule is a chaperone that has been associated with the regulation of TLR-4 membrane localization [53] and could act as a negative regulator of TLR-1 surface trafficking [54]. Thus, because LPS action is mediated by TLR-4, it is thus feasible to speculate that alliin action could modulate this receptor trafficking or its distribution on the membrane, to counteract the LPS stimuli.

On the other hand, in relation with genes downregulated by alliin, we found that the gene with the highest downregulation by alliin was *Dck* (deoxycytidine kinase). It has been described that 3T3-L1-differentiated adipocytes express very low levels when compared with proliferating 3T3-L1 cells [55]; additionally, another two genes related with adipocyte differentiation process, denominated *Aamdc* [56] and Foxp1 [57], were also downregulated. This reflects and confirms that the 3T3-L1 cells were fully differentiated as adipocytes when preincubated with alliin and stimulated by LPS.

In addition, our analysis shows a group of 15 genes that are related with cancer (Table 3). The anticarcinogenic properties of garlic are well documented. Therefore, we present here a list of genes whose expression is downregulated by alliin, which requires more research to unveil their participation in response to alliin treatment and their involvement as anti-carcinogenic factors. Finally, we showed a group of 10 genes that are downregulated by alliin and whose functions remain unknown, suggesting that a relevant portion of alliin effects is yet to be identified.

Taken together, our results may partially explain the effect elicited by alliin; however, further studies looking at the upstream and downstream effector molecules will be necessary in order to understand the role of inflammatory signals in these impairments. Analyses of the roles of certain other genes with as yet unknown functions, the expressions of which were significantly changed, are currently in progress in our laboratory. Additionally, it will be of interest to test the effect of alliin also in immune cells, such as macrophages, or in an *in vivo* study, where macrophage infiltration is present in the adipose tissue. Thus, other yet unknown mechanisms appear to account for the loss of LPS responsibility in adipocytes isolated from inflamed adipose tissue.

## 5. Concluding Remarks

In conclusion, the results presented here demonstrate the possible mechanism by which alliin, a garlic compound, controls the inflammatory state of adipocytes by decreasing IL-6 and MCP-1 expressions (both at mRNA and protein levels), as well as diminishing ERK1/2 phosphorylation in LPS-stimulated 3T3-L1 adipocytes and generating an anti-inflammatory gene expression profile in adipocytes and modifying their metabolic profile.

Additionally, we confirmed that alliin modifies the mRNA expression of genes involved in phospholipid and organophosphate metabolic processes, in positive regulation of the related immune process (expressing immunoglobulin (Ig) and in T-cell receptor-related genes), some enzymes for metabolic and energy process, and genes involved in cancer. All of these processes can be somehow involved in adipocyte protection against a proinflammatory stimulus; thus, they constitute interesting groups of genes to be further explored as involved in adipocyte physiology derived from the alliin action. A deeper understanding of the mechanisms that regulate anti-inflammatory signaling in adipocytes by alliin action may contribute to unraveling possible treatments for obesity-induced inflammation and insulin resistance.

## Supplementary Material

Supplementary materials include four tables. Table 1-SM. Clusters with an enrichment score of >1.5 and representative genes whose expression is significantly modified by LPS in 3T3-L1 differentiated adipocytes. Table 2-SM. Functional categories and genes included whose expression is significantly modified by alliin treatment in 3T3-L1 differentiated adipocytes. Table 3-SM. Genes highly expressed in 3T3-L1 adipocytes by alliin pretreatment and after LPS stimuli. Table 4-SM. Genes that decrease their expression in 3T3-L1 adipocytes by alliin pretreatment and after LPS stimuli.

## Figures and Tables

**Table 1:** Primer sequences used for real-time quantitative PCR.

Gene name	Ensembl gene	Primer sequence (5′-3′)	bp	Tm
*Il-6 *	ENSMUST00000026845	F-TACACATGTTCTCTGGGAAATCGT R-AAGTGCATCATCGTTGTTCATACA	85	75

*Aprt *	ENSMUST00000006764	F-CAGCGGCAAGATCGACTACA R-AGCTAGGGAAGGGCCAAACA	67	60

*Mcp-1 *	ENSMUST00000000193	F-GCTGGAGAGCTACAAGAGGATCA R-CTCTCTCTTGAGCTTGGTGACAAA	79	60

*Tnf*-*α*	ENSMUST00000025263	F-ATGGCCCAGACCCTCACA R-TTGCTACGACGTGGGCTACA	73	60

*Egr-1 *	ENSMUST00000064795	F-GCCGAGCGAACAACCCTAT R-CCATCGCCTTCTCATTATTCAGA	77	60

**Table 2:** Group analysis of genes that increase their expression in 3T3-L1 adipocytes by alliin pretreatment and after LPS stimuli.

		With known function (12 genes 43%)	Precise function unknown (16 genes—57%)
Immune response (10 genes—36%)	Immunoglobulin related		M27752, X53400, AJ400981, X86534, X80972, X66457
	T-cell receptor related		AF041900, AF158133, Z12217, Z86011

Enzymes (8 genes—29%)		Fam213b/C1orf93, Tdh, Acaa1a, Pik3cd, Phka1, Cbs	Cyp2g1, lactate dehydrogenase A-4 pseudogene,

Others (8 genes—29%)	B-cell Chemotaxis	Cxcl13	
	TLR-4 related	Cnpy4	
		Slirp, Tnnt1, Hint2, Nub1	Zfp92, Gm10762

Unclassified (2 genes—7%)			AF357337, AK018247

**Table 3:** Group analysis of genes that decrease their expression in 3T3-L1 adipocytes by alliin pretreatment and after LPS stimuli.

Cancer related (15 genes—43%)	Hoxb13, Hmg20b, Cxcl16, Uimc1, Elf1, Foxp1, Ptgdr, Psmd8, Peg3, Myo15, Lig1,	
	Ptbp2, Kif2c, **Grhpr, Fah**	

Enzymes (5 genes—14%)	Dck, Zdhhc4, Pik3c2g, **Grhpr, Fah**	

Diverse functions (7 genes—20%)	Adipogenesis	Aamdc
	Nuclear	Rny1, Polr1d, Nol12,
	Notch signaling	Dtx2
	Vascular remodeling	Reck/St15
	Ig related	S72845

Unknown function (10 genes—29%)	AK010802, AK007003, AK016406, AK021335, AK015506, AK009275, AK007343,	
	AK006390, AK006570, AK005580	

Bolded gene names correspond to genes that fits into two groups (because are cancer related and also enzymes).
