# Supplementary material for: Alliin, a Garlic (Allium sativum) Compound, Prevents LPS-Induced Inflammation in 3T3-L1 Adipocytes
Source: Mediators Inflamm. 2013 Dec 26;2013:381815. doi: 10.1155/2013/381815 (PMC3888727; doi:10.1155/2013/381815)
Supplement: Supplementary file 1 — Supplementary materials include four tables. Table 1-SM. Clusters with an enrichment score of >1.5 and representative genes whose expression is significantly modified by LPS in 3T3-L1 differentiated adipocytes. Table 2-SM. Functional categories and genes included whose expression is significantly modified by alliin treatment in 3T3-L1 differentiated adipocytes. Table 3-SM. Genes highly expressed in 3T3-L1 adipocytes by alliin pretreatment and after LPS stimuli. Table 4-SM. Genes that decrease their expression in 3T3-L1 adipocytes by alliin pretreatment and after LPS stimuli. [file 381815.f1.pdf]

Table 1-SM

CLS	Enrichment score	Function	p-value	Benjamini	Genes included	Gene examples	Ref
1	3.52	Circulatory system process, vasodilatation	1.70E-04	6.40E-02	7	Angiotensin II receptor,<br>Type 1a neurotensin	Br J Pharmacol. 116(5):2524-30. 1995.<br>BBA. 1813(10):1863-71. 2011.
2	2.80	Thyroglobulin type-1	7.60E-04	7.80E-02	4	Thyroglobulin type-1	Bull Exp Biol Med. 152(2):253-7. 2011
3	2.15	Response to peptide hormone stimulus	8.00E-03	3.40E-01	7	Alpha-2-HS-glycoprotein (AHSG)	Inflammation. 15(5):369-79. 1991.
4	2.06	Methylation	8.30E-03	3.00E-01	8	-	-
5	2.05	Cell motility, migration	3.90E-03	2.90E-01	10	Chemokine (C-C motif) receptor 4<br>Lysosomal trafficking regulator	J Exp Med. 191(10):1755-64. 2000.<br>J Immunol. 166(7):4586-95. 2001.
6	2.01	Cytoplasmic membrane-bounded vesicle	4.60E-03	2.50E-01	14	Angiotensin II receptor,<br>Type 1a neurotensin	Br J Pharmacol. 116(5):2524-30. 1995.<br>BBA. 1813(10):1863-71. 2011.
7	1.90	Respiratory system development	6.80E-03	3.10E-01	7	Cytochrome P450, family 1, subfamily a, polypeptide 2	Neuro Endocrinol Lett.;30 Suppl 1:41-5. 2009.
8	1.79	DNA/RNA helicase, DEAD/DEAH box type, ATP-binding	6.20E-03	7.60E-01	7	HLA-B-associated transcript 1A	DNA Seq.17(4):292-9. 2006
9	1.74	Positive regulation of phagocytosis and vesicle-mediated transport	5.40E-03	3.20E-01	5	Fc receptor, IgG, low affinity III<br>CD209b antigen	PLoS Pathog. 5(6):e1000464. 2009<br>J Immunol. 184(5):2627-37. 2010.
10	1.62	ATP-dependent helicase activity	1.30E-02	4.50E-01	6	HLA-B-associated transcript 1A	DNA Seq.17(4):292-9. 2006

Table 2-SM

Functional category	GENBANK ACCESSION	Gene encoding for
Phospholipid and organophosphate metabolic process	428165	1-acylglycerol-3-phosphate O-acyltransferase 5 (lysophosphatidic acid acyltransferase, epsilon)
	478756	phosphatidylinositol 3-kinase catalytic delta polypeptide
	464983	phosphatidylinositol 3-kinase, C2 domain containing, gamma polypeptide
	424204	phosphatidylinositol glycan anchor biosynthesis, class M
	447430	phosphorylase kinase alpha 1
	435646	receptor-like tyrosine kinase
Positive regulation of immune effector process	425225	forkhead box P1
	474977	immunoglobulin heavy chain 3 (serum IgG2b); (gamma polypeptide)
	453008	retinoic acid early transcript 1E; beta; alpha; delta; gamma
Microtubule, cytoskeleton, motor protein, cell motility	430572	kinesin family member 17
	479264	myosin XV
	426096	similar to Kinesin-like protein KIF2C (Mitotic centromere-associated kinesin) (MCAK)
	450158	tubulin tyrosine ligase-like family, member 9
	451630	deleted in colorectal carcinoma
	435514	laminin B1 subunit 1
	435646	receptor-like tyrosine kinase
	435290	Hermansky-Pudlak syndrome 4 homolog (human)
	443300	thymoma viral proto-oncogene 1 interacting protein
Purine ribonucleotide binding, GTP binding, ATP binding	483211	Era (G-protein)-like 1 (E. coli)
	460221	RIKEN cDNA 1810035L17 gene
	465328	dedicator of cytokinesis 6
	456817	developmentally regulated GTP binding protein 1
	449090	glyoxylate reductase/hydroxypyruvate reductase
	430572	kinesin family member 17
	457106	ligase I, DNA, ATP-dependent
	464176	mitochondrial translational initiation factor 2
	464581	mutS homolog 3 (E. coli)
	479264	myosin XV
	478756	phosphatidylinositol 3-kinase catalytic delta polypeptide
	464983	phosphatidylinositol 3-kinase, C2 domain containing, gamma polypeptide
	461623	polypyrimidine tract binding protein 2
	435646	receptor-like tyrosine kinase
	426096	similar to Kinesin-like protein KIF2C (Mitotic centromere-associated kinesin) (MCAK)
Iron ion binding	474237	cystathionine beta-synthase
	480553	cytochrome P450, family 2, subfamily c, polypeptide 68
	468579	cytochrome P450, family 2, subfamily g, polypeptide 1
Transcription	427656	Kruppel-like factor 16

regulation, zinc finger binding	425225	forkhead box P1
	440843	paternally expressed 3; antisense transcript gene of Peg3
	435860	zinc finger protein 292
	461227	zinc finger protein 92
	431877	bassoon
	430286	deltex 2 homolog (Drosophila)
	452394	zinc finger, DHHC domain containing 4
	478297	calcium channel, voltage-dependent, T type, alpha 1H subunit
	424420	delta-like 4 (Drosophila)
	448999	fumarylacetoacetate hydrolase
	457106	ligase I, DNA, ATP-dependent
	423146	solute carrier family 17 (sodium-dependent inorganic phosphate cotransporter), member 6
	464581	mutS homolog 3 (E. coli)
	437690	ubiquitin interaction motif containing 1
	444913	Crm, cramped-like (Drosophila)
	425239	E74-like factor 1
	460221	RIKEN cDNA 1810035L17 gene
	426391	high mobility group 20 B
	479919	homeo box B13
	458966	polymerase (RNA) I polypeptide D; predicted gene 12751
	472076	nucleolar protein 12
Apoptosis	451630	deleted in colorectal carcinoma
	476880	histidine triad nucleotide binding protein 2
	440843	paternally expressed 3; antisense transcript gene of Peg3
	443300	thymoma viral proto-oncogene 1 interacting protein
Cytoplasmic membrane-bounded vesicle	435290	Hermansky-Pudlak syndrome 4 homolog (human)
	461196	proacrosin binding protein
	423146	solute carrier family 17 (sodium-dependent inorganic phosphate cotransporter), member 6
	431877	bassoon
	468924	paraoxonase 1
	435646	receptor-like tyrosine kinase
	436778	reversion-inducing-cysteine-rich protein with kazal motifs precursor (mRECK)
Integral to membrane	428165	1-acylglycerol-3-phosphate O-acyltransferase 5 (lysophosphatidic acid acyltransferase, epsilon)
	480226	acyl-malonyl condensing enzyme 1
	478297	calcium channel, voltage-dependent, T type, alpha 1H subunit
	482929	chemokine (C-X-C motif) ligand 16
	451630	deleted in colorectal carcinoma
	424420	delta-like 4 (Drosophila)
	474977	immunoglobulin heavy chain 3 (serum IgG2b); Immunoglobulin heavy chain (gamma polypeptide)

424204	phosphatidylinositol glycan anchor biosynthesis, class M
445826	prostaglandin D receptor
435646	receptor-like tyrosine kinase
453008	retinoic acid early transcript 1E; beta; alpha; delta; gamma
435318	retrotransposon-like 1; RIKEN cDNA 6430411K18 gene
436778	reversion-inducing-cysteine-rich protein with kazal motifs precursor (mRECK)
423146	solute carrier family 17 (sodium-dependent inorganic phosphate cotransporter), member 6
441284	solute carrier family 35, member B4
456728	solute carrier family 7 (cationic amino acid transporter, y+ system), member 9
434029	transmembrane protein 14A
421600	transmembrane protein 81
452394	zinc finger, DHHC domain containing 4

Table 3-SM

Genes highly expressed in 3T3-L1 adipocytes by alliin pretreatment and after LPS stimuli

Genbank	Gene name	z-score
AF041900	T-cell receptor beta chain	3.40
NM_009566	Zfp92	3.20
M27752	Ig rearranged H-chain V-region	3.05
M17518	lactate dehydrogenase A-4 pseudogene	2.96
X53400	Igh-V7183	2.94
AF357337	unclassified gene	2.90
AJ293625	Slirp	2.85
AF158133	T-cell receptor beta chain V-D-J region	2.81
NM_013809	Cyp2g1	2.78
AJ400981	Igh-VI55H	2.78
NM_018866	Cxcl13	2.76
U43721	Cbs	2.75
NM_025582	Fam213b / C1orf93	2.75
AK011474	Cnpy4	2.72
Z12217	T-cell receptor beta chain Vbeta8 (VDJ)	2.71
X86534	Igh-VH10B8	2.71
AK014355	unclassified gene	2.68
NM_021480	Tdh	2.68

Z86011	T-cell receptor, V-J beta region	2.67
NM_011618	Tnnt1	2.65
X80972	Igh-gamma polypeptide	2.63
BC012400	Acaa1a	2.63
NM_008840	Pik3cd	2.62
NM_008832	Phka1	2.59
AK018247	unclassified gene	2.58
AK004497	Hint2	2.53
X66457	Igh-V10C5	2.52
NM_016736	Nub1	2.52

Table 4-SM

Genes that decrease their expression in 3T3-L1 adipocytes by alliin pretreatment and after LPS stimuli

Genbank	Gene name	z-score
NM_007832	Dck	-6.19
NM_008267	Hoxb13	-5.23
AF146224	Hmg20b	-5.18
AF301017	Cxcl16	-3.72
AK010802	unclassified gene	-3.60
U34828	Rny1	-3.55
S72845	Ig V lambda 1 variable region	-3.51
NM_009087	Polr1d	-3.44
U22015	Uimc1	-3.41
AK007003	unclassified gene	-3.28
AK013609	Zdhhc4	-3.26
AK016406	unclassified gene	-3.26
NM_007920	Elf1	-3.22
AK021335	Thyn1	-3.20
AF339103	Foxp1	-3.17
NM_008962	Ptgdr	-3.12
BC013701	Nol12	-3.09

NM_026545	Psmd8	-3.07
AK015506	unclassified gene	-3.02
AK018293	Grhpr	-3.02
NM_010862	Myo15	-2.98
AB015423	Dtx2	-2.97
NM_016678	Reck	-2.97
NM_010176	Fah	-2.89
AK009275	Commd8	-2.87
L10896	Peg3	-2.87
NM_010715	Lig1	-2.84
AK007343	Hsbp1l1	-2.82
NM_019550	Ptbp2	-2.76
BC006841	Kif2c	-2.67
AK006390	unclassified gene	-2.65
AK006570	unclassified gene	-2.61
NM_011084	Pik3c2g	-2.59
AK005580	unclassified gene	-2.59
AK008195	Aamdc	-2.59
